# Sternoclavicular Septic Arthritis: A Case Report

**DOI:** 10.7759/cureus.38130

**Published:** 2023-04-25

**Authors:** Michael Cydylo, Ivan Ivanov, Jessica Chineme

**Affiliations:** 1 Emergency Department, New York City Health and Hospitals - South Brooklyn Health, Brooklyn, USA

**Keywords:** emergency medicine, staph aureus, trimethoprim-sulfamethoxazole, patient autonomy, sternoclavicular joint (scj) septic arthritis

## Abstract

We report a case of a 47-year-old male who presented with concerns for a "mass" on the right side of his chest and low-grade fevers for the last month. The patient was found to have an induration, erythema, and warmth at the right sternoclavicular joint, with tenderness to palpation and pain in the movement of the right arm. The patient was found to have septic arthritis of the sternoclavicular joint based on CT imaging. Sternoclavicular joint septic arthritis is a rare diagnosis and accounts for very few diagnosed septic joints. Most patients have some sort of risk factors, such as diabetes, immunosuppression, rheumatoid arthritis, or intravenous drug use. The most common pathogen is *Staphylococcus aureus*. Our patient did not consent to joint aspiration for a definitive diagnosis of the causative organism and was therefore empirically treated for *S. aureus* with trimethoprim-sulfamethoxazole. The patient also did not consent to any surgical management. Septic arthritis has been successfully treated with antibiotic therapy alone in the past, and in conjunction with the patient’s choices, this was the treatment plan that was chosen for the patient. The patient responded to antibiotic therapy and followed up with a thoracic surgery clinic outpatient. This case depicts the importance of retaining a high index of suspicion for a rare diagnosis in the emergency department (ED). This case also depicts the successful treatment of sternoclavicular septic arthritis with outpatient oral trimethoprim-sulfamethoxazole, which, to the best of our knowledge, has not been done previously.

## Introduction

Infection of the sternoclavicular joint is a rare diagnosis, accounting for less than 1% of all osseous infections [1]. The majority of patients who develop such a pathology have one of the known risk factors, including immunosuppression, diabetes, intravenous drug use, and rheumatoid arthritis [1-2]. It is also known, however, that up to 25% of patients who develop it have no known risk factors [1-2]. Sternoclavicular joint septic arthritis usually occurs from hematogenous spread, especially in IVDU [3]. Almost half of the infections are caused by Staphylococcus aureus, followed distantly by *Pseudomonas aeruginosa* (10%) and *Brucella melitensis* (7%) [2,4]. A septic sternoclavicular joint should be considered in patients presenting with localized tenderness, erythema, edema, or pain on movement of the sternoclavicular joint [1,5]. Diagnostically, CT imaging shows a sensitivity of 83% for diagnosing sternoclavicular septic joint arthritis and is the preferred imaging modality for diagnosis, while CT-guided aspirations only reveal positive cultures in 52% of cases [6-9]. However, the gold standard of diagnosis remains an arthrocentesis of the joint [1]. A positive culture is diagnostic of an infected sternoclavicular joint, regardless of the other synovial fluid analyses [1]. Blood cultures are positive in over 50% of patients with confirmed infections [9].

Analgesia and broad-spectrum antibiotics should be initiated in the emergency department (ED); however, the gold-standard treatment is surgical debridement and washout with individually tailored antibiotic therapy [1,2,8,9]. Untreated septic joints carry up to 10% mortality with morbidity over 30%, including a prevalence of abscess formation as high as 80% [9,10]. Other complications of sternoclavicular infection include post-surgical complications, osteomyelitis (especially if not treated), joint instability, sepsis, the need for joint reconstruction surgery, the need for repeat surgery, and disseminated infection to the chest wall and mediastinum [5,9,11]. We present a case where a 47-year-old male presented with swelling of the right sternoclavicular joint with fever with an insidious onset that was diagnosed solely with CT and managed with IV antibiotics that were transitioned to outpatient oral trimethoprim-sulfamethaxole therapy.

## Case presentation

A 47-year-old male presented to our ED complaining of one month of right-sided "shoulder" pain. The patient stated that he went to see his primary care physician six days ago because of worsening pain and was started on 20 mg of prednisone twice daily and 400 mg of ibuprofen every six hours as needed, which the patient states he was taken appropriately. He was instructed to get a CT scan but was unable to make the appointment, so he came to the ED due to severe worsening pain. He also complained of a "bump" growing by his right clavicle and intermittent fevers for the past month. The patient noted that he was experiencing night sweats but attributed them to the lack of air conditioning in his apartment. He stated that he was imprisoned in Belarus for about two to three years prior to immigrating to the United States and had negative tuberculosis testing upon immigration. The negative tuberculosis test was four months prior to his presentation to the emergency department. The patient denied weight changes, intravenous drug use, any history of sexually transmitted diseases, or an immunocompromised state. The patient is sexually active with his wife and no other partners, but he does not use protection.

**Figure 1:**
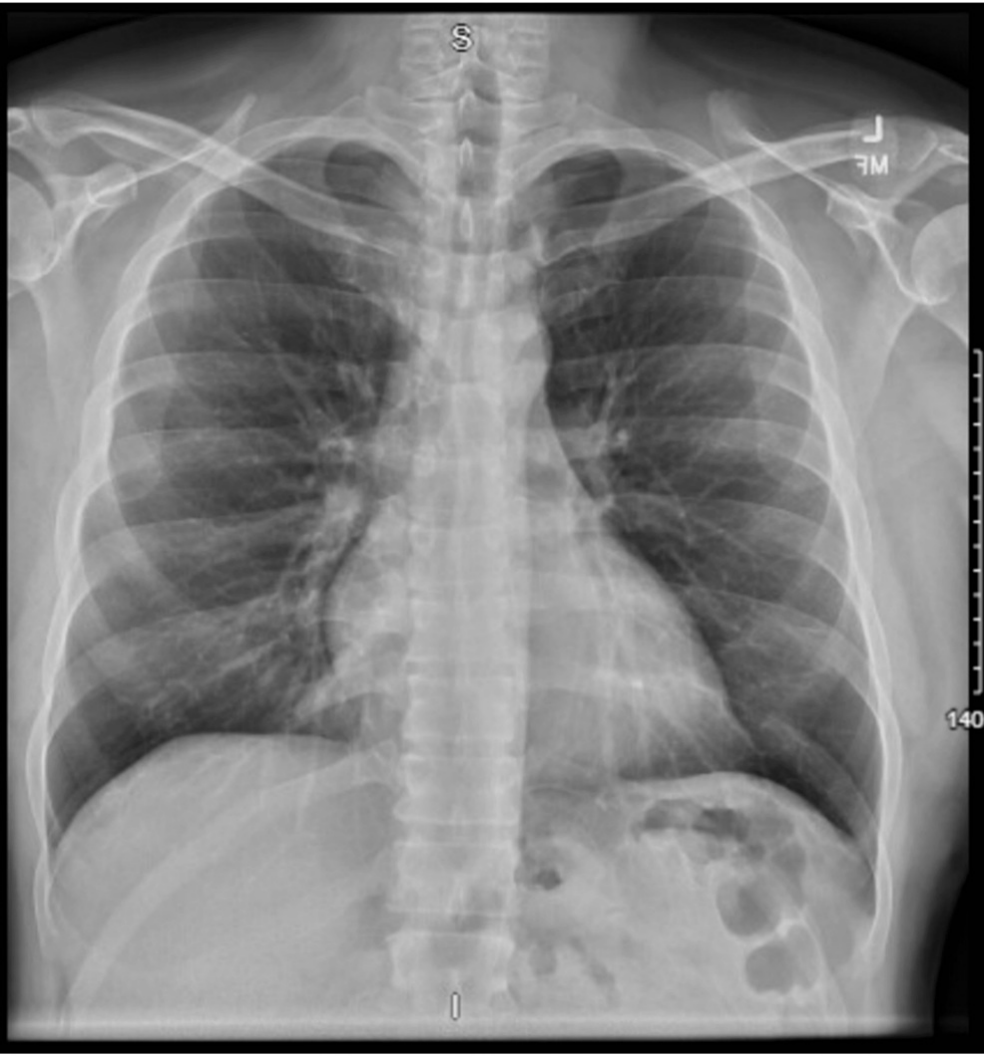
Chest X-ray - no acute pathology

**Figure 2:**
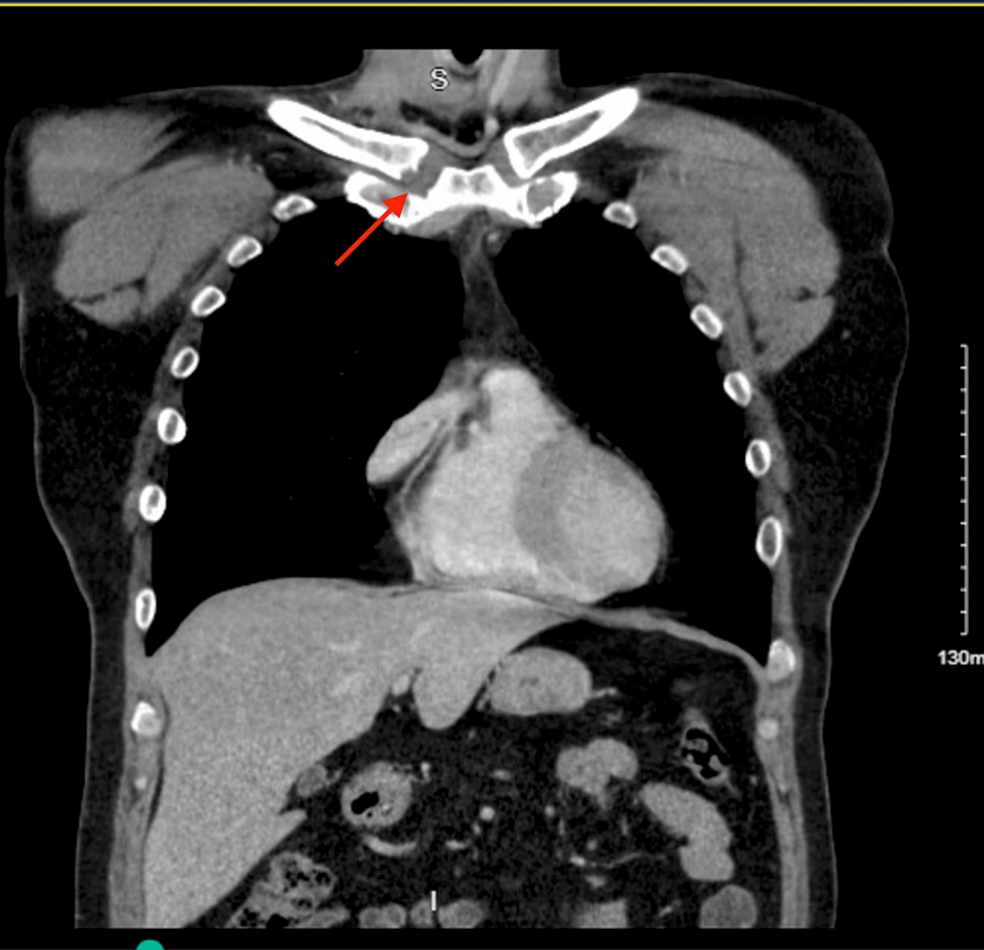
CT chest with IV contrast, coronal view: demonstrating induration and subcutaneous fat stranding at the right sternoclavicular joint with evidence of destruction of the cortical portion of the clavicle. There also appears to be increased soft tissue density within the joint.

**Figure 3:**
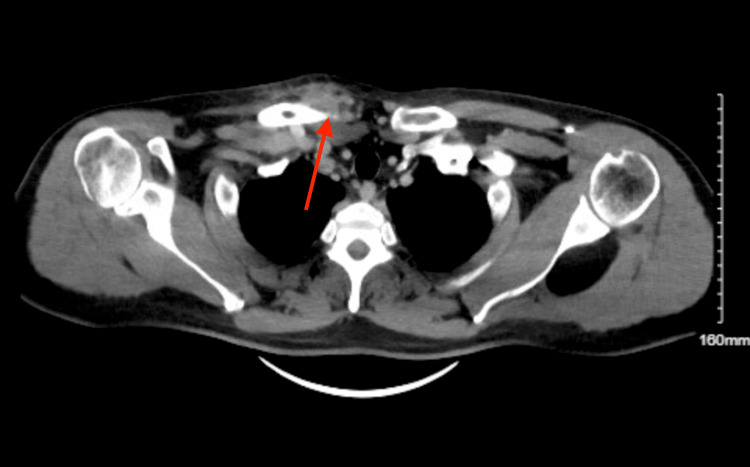
CT chest with IV contrast, transverse view: further demonstrates the same findings.

On presentation, the patient’s vital signs were stable with a blood pressure of 157/100 mmHg, a heart rate of 75 bpm, afebrile at 98.6 °F, and a respiratory rate of 18 saturating at 100% on room air. The patient had a warm, indurated mass at the right sternoclavicular joint and complained of severe pain with range of motion testing of his right arm, especially on adduction. Initial labs showed a non-elevated white count of 9.41 × 103/mcL with no left shift, a non-reactive HIV antigen and antibody screen, and a negative urine drug screen for opiates, methadone, cocaine, benzodiazepines, and barbiturates. Significant labs were an elevated erythrocyte sedimentation rate of 55 mm/hr (normal 0-15 mm/hour) and an elevated C-reactive protein at 52.8 mg/L (normal 0-5 mg/L). The initial chest X-ray is shown in Figure 1 and showed no acute pathology. A CT chest with intravenous (IV) contrast was performed and is shown in Figures 2-3, which showed induration and stranding of the subcutaneous fat with cortical destruction of the clavicular portion of the joint. The patient was started on 1500 mg of intravenous vancomycin and 3.375 g of intravenous piperacillin-tazobactam in the ED and then was admitted to the surgical team for further management. The patient’s quantiferon later returned and was indeterminate; however, our infectious disease specialist had low concern for tuberculosis as the causative agent. The patient had gonorrhea and chlamydia tests that also returned the next day and were negative. Blood cultures showed no growth of any organisms. Echocardiography was performed while the patient was admitted and showed no acute findings. We initially did consider a possible rheumatological process; however, the patient did not respond to outpatient prednisone, and CT findings were consistent with an infective process. Other infectious etiologies like gonorrhea and tuberculosis were considered and ruled out; therefore, broad-spectrum antibiotics were continued. While admitted, the patient’s swelling and pain mostly resolved, and the patient did not consent to joint aspiration for a definitive diagnosis of the pathological organism or any surgical management. The patient’s presumed pathogen was *S. aureus*, so the patient was started on 160-800 mg of trimethoprim-sulfamethoxazole twice daily and ibuprofen 400 mg every six hours as needed for pain with further improvement of symptoms and then discharged. The patient followed up in the surgery clinic after one month of trimethoprim-sulfamethoxazole and the swelling, warmth, and pain had almost entirely resolved. The patient was continued on another month of trimethoprim-sulfamethoxazole."

## Discussion

There are no diagnostic criteria for diagnosing a septic sternoclavicular joint. A diagnosis is obtained through a thorough history, physical, laboratory workup, imaging modalities, and arthrocentesis if possible. Sternoclavicular joint infections are mostly unilateral, in fact mostly on the right (60%), and can present with fever, joint swelling, warmth, and immobility [6]. CT-guided arthrocentesis only provides a positive culture in a little over half of cases; therefore, CT imaging is an important part of the workup and can be diagnostic [7,8]. Our patient presented with a history, physical, and CT findings that were consistent with sternoclavicular joint septic arthritis.

There have been very few documented cases of sternoclavicular septic arthritis treated only with antibiotics and no surgical intervention [3,12,13]. Renoult reports two cases of bacterial sternoclavicular septic arthritis in hemodialysis patients, both of whom were successfully treated with directed antibiotic therapies. They suggest that chronic indwelling ports, such as those for hemodialysis, increase the risk of a septic joint [12].

Zanelli et al. report a patient without known risk factors who was treated with antibiotic therapy alone for a confirmed *S. aureus* infection of the sternoclavicular joint [13]. Risk factors they screened for include immunosuppression, diabetes, alcoholism, connective tissue disorders, intravenous substance abuse, trauma, arthritic conditions, and subclavian venous access. The patient was successfully treated with a four-week course of intravenous teicoplanin and ceftriaxone despite presenting with early signs of osteomyelitis, a late finding in the disease process [13].

While surgical intervention alongside targeted antibiotic therapy is the gold standard of treatment, there have been several cases of confirmed sternoclavicular septic arthritis successfully treated with antibiotic therapy alone [2,5,12,13]. Aggressive surgical management should be pursued if there is extensive bony destruction, osteomyelitis, chest wall or retrosternal abscess, mediastinitis, or pleural extension [3]. However, in cases of less extensive disease, medical therapy alone or with limited surgical intervention has proven to be effective [3]. In the recent external validation of the OVIVA trial in 2020, oral antibiotics were shown to be as efficacious as intravenous antibiotics for the treatment of bone and joint infections [14]. We report an additional case of uncomplicated sternoclavicular septic arthritis that improved with outpatient oral trimethoprim-sulfamethoxazole after one month, then repeated for a second month due to incomplete full resolution.

Surgical intervention, while the gold standard treatment, also carries associated morbidity not seen with antibiotic therapy. Adverse reactions to anesthesia, post-surgical infections, and the need for additional surgeries, for example, must be weighed when considering surgical treatment. In patients without severe signs of osteomyelitis or systemic infection, treatment with antibiotics alone should be considered a treatment option. Some limitations to our case were the inability to perform arthrocentesis due to patient preference, and therefore we were unable to guide antibiotic therapy toward a specific pathogen. Another limitation was the inability to go for surgical debridement due to patient preference, which would have shown the full extent of the infection.

## Conclusions

As an emergency medicine physician, keeping the differential diagnosis of sternoclavicular joint septic arthritis in mind when patients present with unilateral pain near their sternoclavicular joint is critical in order to prevent late-stage complications. In patients who do not accept diagnostic algorithms, early broad-spectrum antibiotic therapy protects patients from morbidity. Although a surgeon should also be involved early, some of these patients can be treated with antibiotic therapy alone. Patient autonomy is an important factor to keep in mind when deciding on any treatment plan.
